# Moving towards better cause of death registration in Africa and Asia

**DOI:** 10.3402/gha.v7.25931

**Published:** 2014-10-29

**Authors:** Ties Boerma

**Affiliations:** Health Statistics and Information Systems, World Health Organization, Geneva, Switzerland

Mortality statistics by age, sex, and cause of death are the foundations of public health programmes. They are essential for planning, programming, and monitoring progress. Ideally, mortality statistics should be available for small areas, to detect differences in the distribution of mortality risks and plan health programmes accordingly. Yet, the majority of countries in sub-Saharan Africa and Asia do not have reliable mortality statistics by age, sex, and cause.

The main reason is that national civil registration and vital statistics (CRVS) systems function poorly in most low- and middle-income countries. Only a small proportion of deaths are registered and reported. An even smaller proportion of deaths is medically certified with a cause of death using the World Health Organization's International Classification of Diseases (ICD).

Special efforts are needed to fill this critical information gap in countries. Some countries have succeeded in improving cause of death data that are generated in hospitals. This is an important starting point but will only partially address the information deficit as in most countries less than one-fifth of deaths occur in hospitals. The cause patterns of hospital deaths are also likely to differ from those in the community. Household surveys can provide an important vehicle for collecting retrospective information on mortality levels, trends, and differentials. Verbal autopsies (carrying out interviews with witnesses of deaths to estimate likely causes) provide a strategy for documenting deaths that are not otherwise certified. Some countries have included verbal autopsy modules in household surveys to obtain a general picture of the causes of death.

Local health and demographic surveillance system (HDSS) sites are another important source of mortality statistics. Their strength lies in the quality of the information generated and the measurement of trends over time. This volume of *Global Health Action* presents a unique collection of verbal autopsies conducted in 22 different HDSS sites that are part of the INDEPTH Network in 13 countries in sub-Saharan Africa and south and south-east Asia. To my knowledge, the combined total of almost 100,000 verbal autopsies, mostly conducted since 2006, presents the largest data set of this nature ever and provides a nice overview of leading causes of death in mostly rural settings in low- and middle-income countries.

In 2006, a predecessor of this set of papers was published, based on 38,000 verbal autopsies from 12 INDEPTH sites (1). At the time, considerable resources had to be spent to maximise the comparability of the data between study sites, and physicians were used to ascertain the probable cause of death. In this volume of *Global Health Action*, the application of the WHO 2012 verbal autopsy standard (2) and the use of automated assignment of cause of death using the InterVA-4 model (3) ensure more comparable and consistent results. In addition, the replicability and opportunities for further analyses are enhanced by the recent commendable actions of the INDEPTH Network to put cause of death and other data sets into the public domain.

The work of INDEPTH on mortality and causes of death needs to be considered in the context of the need to strengthen country CRVS systems. Increasingly, it is realised that strong CRVS systems are critical for good governance, establishing people's rights and those of their children. It should be a central element in the post-2015 development agenda. The health sector would be a beneficiary of complete and reliable CRVS systems, but it also needs to make a substantial contribution to the recording and reporting of events in a CRVS system (4). The HDSS sites should play important roles in strengthening of CRVS systems. This includes the sharing of their experiences with often innovative methods of data collection on vital events, such as the use of verbal autopsy or technologies; the national capacity strengthening related to generating vital statistics; and the assessment of completeness of an expanding national CRVS system within HDSS populations. This special collection of papers on mortality and causes of death using verbal autopsy presents a significant example of how INDEPTH HDSS sites can contribute to stronger CRVS systems in countries.

*Ties Boerma* Director Health Statistics and Information Systems World Health Organization Geneva, Switzerland
